# Advancing chronic obstructive pulmonary disease management: The case for multi-biomarker precision medicine—A narrative review

**DOI:** 10.1177/27558428261452294

**Published:** 2026-08-25

**Authors:** Anusha Sundararaman, Thangavel Mahalingam Vijayakumar, Nalini Jayanthi Nagesh

**Affiliations:** 1 Department of Pharmacy Practice, SRM College of Pharmacy, Faculty of Medicine and Health Sciences, SRM Institute of Science and Technology, Kattankulathur, Chengalpattu, Tamil Nadu, India; 2 Department of Respiratory Medicine, SRM Medical College Hospital, Faculty of Medicine and Health Sciences, SRM Institute of Science and Technology, Kattankulathur, Chengalpattu, Tamil Nadu, India

**Keywords:** COPD, precision medicine, biomarkers, inflammation, therapy guidance

## Abstract

Chronic obstructive pulmonary disease (COPD) is increasingly recognized as a biologically heterogeneous disorder that extends beyond eosinophilic inflammation alone. Although blood eosinophil counts are clinically useful for guiding inhaled corticosteroid therapy, they represent only a single inflammatory endotype and fail to capture the full spectrum of disease mechanisms that influence progression, exacerbations, and treatment response. This narrative review synthesizes emerging evidence supporting a multidomain, multi-biomarker framework for COPD phenotyping that integrates systemic inflammatory markers, epithelial injury proteins, structural imaging parameters, and environmental or exposomic indicators. Studies published between January 2010 and September 2024 demonstrate that combined assessment of biomarkers such as C-reactive protein, interleukin-6, fibrinogen, surfactant protein-D, club cell secretory protein-16, quantitative computed tomography metrics, and selected exposomic measures improves prediction of exacerbations and mortality compared with eosinophil-guided strategies alone. Advanced approaches that incorporate artificial intelligence and multi-omics data further enhance phenotype identification in research and early translational settings, although routine clinical implementation remains limited by cost, standardization, and feasibility. Overall, movement toward a multi-biomarker precision-medicine paradigm has the potential to refine therapeutic decision-making, reduce inappropriate corticosteroid exposure, and support more equitable, data-driven COPD management. However, successful translation will require robust validation, standardized assays, cost-effective diagnostic platforms, and integration into digital clinical workflows, particularly in low- and middle-income healthcare settings.

## Introduction

Chronic obstructive pulmonary disease (COPD) is a major global health challenge, currently affecting over 300 million people and ranking among the top three causes of death worldwide.^1,2^ While spirometry remains the cornerstone of diagnosis, it inadequately captures the clinical, structural, and biological heterogeneity that defines COPD.^3–5^ The disorder encompasses diverse inflammatory and pathological endotypes influenced by genetics, airway remodeling, comorbidities, and environmental exposures such as tobacco smoke and biomass fuel.^6–8^ This heterogeneity contributes to variable treatment responses and clinical outcomes, underscoring the need for precision-based management strategies.^9–11^

Among available biomarkers, blood eosinophil count has become the most widely adopted surrogate for guiding inhaled corticosteroid (ICS) therapy. Landmark clinical trials, including IMPACT, ETHOS, and BOREAS, established that elevated blood eosinophil levels (⩾150–300 cells/µL) predict responsiveness to ICS or biologic agents targeting the interleukin-4 (IL-4) and interleukin-5 (IL-5) pathways.^2,12,13^ These pivotal findings have been incorporated into the 2024 Global Initiative for Chronic Obstructive Lung Disease (GOLD) guidelines, which recommend eosinophil-based escalation of therapy in patients with frequent exacerbations.^14^ However, longitudinal and real-world studies have shown that eosinophil concentrations vary substantially over time, and only 60%–70% of patients maintain stable eosinophil counts across repeated measurements.^15–17^ Moreover, approximately two-thirds of COPD cases exhibit non-type 2 (neutrophilic or macrophage-dominant) inflammation that is largely corticosteroid-resistant.^18–21^ Consequently, reliance on eosinophils alone risks both overtreatment and undertreatment, particularly in patients with infection-driven or structural airway disease.

Earlier reviews have highlighted the limitations of single-marker approaches and advocated for multidomain biomarker integration, encompassing inflammatory, imaging, microbial, and environmental factors.^6–8,22^ Emerging evidence supports that combining systemic proteins like C-reactive protein (CRP), IL-6, fibrinogen, epithelial markers like surfactant protein -D (SP-D), club cell secretory Protein 16 (CC-16), and quantitative imaging metrics like parametric response mapping (PRM), and mucus-plug scoring yields superior prediction of exacerbations and mortality.^11,19,23,24^ Against this backdrop, this review explores how a multi-biomarker precision medicine framework can redefine COPD phenotyping and therapeutic decision-making, advancing the transition from guideline-based to individualized care.

## Methodology

This narrative review was undertaken to examine the constraints of eosinophil-guided therapy and to assess the developing function of multi-biomarker panels in directing contemporary pharmacological targets for COPD, especially regarding personalized triple therapy and biologics. This article is a narrative expert review and does not constitute a systematic review or meta-analysis.

A comprehensive literature search was conducted in PubMed, Scopus, Embase, Web of Science, and Google Scholar databases for studies published between 2010 and 2024. The search focused on clinical trials, systematic reviews, cohort studies, and guideline-based recommendations addressing biomarkers and drug targets in COPD.

The following key terms were used individually and in Boolean combinations: “*COPD*,” “*biomarkers*,” “*eosinophils*,” “*non-T2 inflammation*,” “*IL-5*,” “*IL-6*,” “*IL-33*,” “*TNF-α*,” “*biologics in COPD*,” “*triple therapy*,” “*benralizumab*,” “*dupilumab*,” “*tocilizumab*,” “*itepekimab*,” “*surfactant protein D*,” “*mucus plugs*,” “*PRM*,” “*biomass exposure*,” “*anti-inflammatory targets*” and “*targeted therapy in COPD.*”

The identified literature was thematically synthesized into biomarker categories linked to their corresponding therapeutic targets. A mechanistic mapping approach was employed to correlate biomarkers (e.g., eosinophils, IL-6, SP-D, 8-isoprostane, IgE) with current and investigational drug therapies, highlighting their roles in COPD endotyping and precision treatment pathways.

This narrative review was conducted and reported in accordance with the SANRA (Scale for the Assessment of Narrative Review Articles) guidelines (Supplemental File).^25^

## Nature and limitations of the review

This article is a narrative expert review intended to synthesize and contextualize evolving evidence on biomarker-guided precision medicine in COPD. As a narrative review, no formal risk-of-bias or quality-assessment tools were applied. Potential selection bias cannot be excluded, as study inclusion was based on thematic relevance and translational significance rather than predefined inclusion criteria. In addition, narrative synthesis makes it difficult to directly compare effect sizes across studies and may place greater emphasis on exploratory or hypothesis-generating findings. Consequently, several biomarker-based strategies discussed should be interpreted as research-phase or early translational approaches that require further prospective validation before routine clinical adoption.

## Emerging multi-biomarker paradigm

While several multi-biomarker, imaging-based, and artificial intelligence-driven approaches discussed in this review demonstrate strong biological plausibility and promising performance in observational cohorts and early trials, many remain in the research or early translational phase. These strategies should therefore be interpreted as adjunctive or investigational tools, pending further prospective validation, standardization, and assessment of feasibility before routine clinical adoption. Recognizing the multifactorial nature of COPD, recent translational research emphasizes a multi-biomarker approach that integrates inflammatory, immunologic, structural, microbial, and environmental indicators.^11,19,22,26,27^ These composite profiles enable improved endotyping, guide therapy selection, and enhance prediction of exacerbations, disease progression, and mortality.^11,19,23^

### Integrated inflammatory and systemic panels

Combinations of systemic markers such as CRP, IL-6, and fibrinogen, alongside epithelial-derived proteins including SP-D and CC-16, provide a broader reflection of airway and systemic inflammation.^19,23,28^ Elevated CRP (>3 mg/L) and fibrinogen levels are independently associated with frequent exacerbations and all-cause mortality.^19,29^ The ECLIPSE and SUMMIT cohorts demonstrated that combining these markers with imaging features improves prognostic accuracy for future exacerbations compared with single-marker approaches.^19,23,24^ SP-D and CC-16, as markers of alveolar integrity and epithelial injury, correlate with emphysema severity and progression, thus bridging inflammatory activity and structural damage.^23,24,28^

### Radiologic and structural integration

Advances in quantitative computed tomography (CT) enable the extraction of imaging biomarkers such as PRM, mucus-plug quantification, and airway-wall thickness (Pi10), which differentiate functional small-airway disease from emphysema.^20,24,30–32^ PRM has been shown to predict mortality independently of lung function or eosinophil count.^20,24^ The COPDGene study demonstrated that integrating CT metrics with systemic biomarkers such as SP-D and IL-6 substantially improves phenotypic precision and outcome prediction.^20,24,33^ Machine-learning algorithms trained on multi-center CT datasets now automate pattern recognition and allow high-throughput phenotyping and reproducible biomarker and imaging integration.^33–36^ Although quantitative CT–derived biomarkers such as PRM and mucus-plug quantification provide important structural and prognostic information in COPD, their use in routine longitudinal follow-up is limited by cumulative radiation exposure.^21,24,37^ In addition, cost and infrastructure requirements restrict widespread implementation of advanced imaging-based biomarkers, particularly in low- and middle-income healthcare settings.^38^ Accordingly, CT-based biomarkers should be considered adjunctive tools for selected clinical indications rather than routine monitoring instruments, with greater emphasis placed on scalable blood-based and non-invasive biomarkers for longitudinal assessment.^37,39^

### Microbial and exhaled biomarkers

The airway microbiome adds an additional layer of pathophysiologic insight and represents a relatively under-integrated biomarker domain in current precision approaches to COPD. 16S rRNA sequencing of sputum samples has identified pathogen-predominant phenotypes associated with frequent exacerbations, neutrophilic inflammation, and diminished ICS responsiveness, particularly within asthma–COPD overlap (ACO) populations, primarily in observational and translational studies.^22,40,41^ Shifts in bacterial composition correlate with inflammatory cytokine signatures, particularly IL-8 and tissue necrosis factor (TNF-α).^40^ Non-invasive exhaled biomarkers, including fractional exhaled nitric oxide (FeNO), volatile organic compounds (VOCs), extracellular vesicles (EVs), and 8-isoprostane, serve as surrogates of airway inflammation, oxidative stress, and epithelial activation.^42–48^ Elevated FeNO (>25 ppb) identifies corticosteroid-responsive phenotypes, whereas high 8-isoprostane levels indicate oxidative injury linked to biomass exposure.^6,8,49–51^ Recent clinical evidence further suggests that respiratory microbiome dysbiosis may influence disease activity and therapeutic responsiveness in ACO, supporting its potential role as a missing biomarker in precision COPD frameworks.^52,53^ Despite this biological relevance, lung microbiome profiling currently remains a research-phase or early translational tool, limited by variability in sampling methods, sequencing platforms, and lack of standardized clinical thresholds.

### Environmental and exposomic dimensions

In low- and middle-income countries (LMICs), where biomass fuel and air pollution are major etiologic drivers, environmental biomarker integration is essential. Biomass-related COPD is typically neutrophil-dominant, with elevated CRP, SP-D, TNF-α, and oxidative-stress markers.^6,8,49–51^ Composite panels incorporating these markers demonstrate improved sensitivity for exacerbation prediction in non-eosinophilic populations.^6,8,26^ The inclusion of real-time exposure data from wearable sensors and satellite-derived air-quality indices within biomarker models offers opportunities for dynamic monitoring and early intervention.

### Artificial intelligence and multi-omics integration

Modern artificial intelligence (AI) frameworks integrate proteomic, metabolomic, transcriptomic, and microbiomic datasets with clinical and imaging parameters to build predictive models of disease trajectory.^22,33,35,54,55^ Deep-learning systems trained on COPDGene and ECLIPSE cohorts have shown potential to improve prediction of ICS responsiveness and mortality compared with traditional indices, primarily in research and early translational settings.^33,34,56^ While these approaches show considerable promise, their current role is best viewed as investigational decision-support tools, representing an important step toward but not yet achieving scalable, real-world precision medicine in COPD.

Below, we detail each biomarker category, including their advantages and limitations, which is depicted in Table 1 (advantages, limitations, validation) and Table 2 (ranking biomarkers by clinical applicability).

**Table 1.** Emerging biomarkers in COPD: advantages, limitations, and validation.

Biomarker type	Examples	Advantages	Limitations	Validation status
Inflammatory	CRP, IL-6, fibrinogen, IL-33	These biomarkers have a high prevalence in routine clinical practice. They are inexpensive and serve as robust predictors of exacerbation risk and mortality, as demonstrated in the SUMMIT and ECLIPSE studies.	They lack specificity, are frequently elevated during infections or comorbid states, and cannot distinguish among COPD subtypes.	Validation is high, as these biomarkers have been confirmed in randomized controlled trials and large cohorts, showing an area under the curve (AUC) greater than 0.8 and high inter-assay reproducibility.
Immunological	IgE, IgA, and IgG subclasses	These biomarkers are useful in identifying overlap syndromes such as asthma–COPD overlap (ACO), detecting humoral immune deficits, and stratifying atopic burden.	Their relevance is variable outside the asthma–COPD overlap context, and they have limited external validation with overlap in allergic conditions.	Validation is moderate and supported by specific ACO-focused cohorts and selected multi-cohort trials.
Microbial	Sputum cultures, 16S rRNA sequencing	These biomarkers improve pathogen detection, guide antibiotic stewardship, and are essential for defining infection-related COPD phenotypes.	Their interpretation depends on sampling technique sensitivity, carries a risk of contamination, and involves high costs when advanced genomic approaches are used.	Validation is moderate, with promising results, but requires robust trials assessing routine clinical use and cost-effectiveness.
Exhaled	FeNO, VOCs, breathomics	These biomarkers are non-invasive, rapid, predictive of type 2 inflammation and early exacerbations, and correlate well with the eosinophilic phenotype.	Their accuracy is device- and platform-dependent, they lack standardization, and there is significant variability between laboratories.	Validation is moderate, with a growing body of device-dependent evidence and ongoing feasibility studies.
Serum	SP-D, sRAGE, and VEGF	These biomarkers reflect epithelial injury, vascular remodeling, and disease progression, and they have high specificity for lung-origin pathology.	They are expensive, subject to assay-dependent variability, and currently limited to use in specialty centers.	Validation is high, supported by robust evidence from the ECLIPSE, COPDGene, and multinational studies, with an AUC greater than 0.75.
CT Imaging-based	PRM, mucus plug quantification, Pi10	These imaging-based biomarkers enable direct quantification of structural lung pathology, objective monitoring of disease progression, and accurate phenotype assignment.	They are resource-intensive due to cost and radiation exposure, and access is restricted outside higher-income healthcare settings.	Validation is high, supported by strong evidence from quantitative imaging trials and longitudinal cohort studies.
Environmental	8-isoprostane, TNF-α	These biomarkers enable precision phenotyping for biomass exposure and provide reliable indices of oxidative stress.	They require specialized laboratory facilities and have been validated only in selected real-world biomass exposure cohorts.	Validation is emerging, with early studies focusing on populations in high-exposure, low- to middle-income settings.
Multi-omics	Proteomics, metabolomics, virome	These biomarkers enable integrative disease modeling and support the development of artificial intelligence and machine learning phenotype predictors.	They remain experimental due to high cost and complexity, are limited to small pilot studies, and currently lack external validation.	Validation is emerging, based on pilot and proof-of-concept studies, and these biomarkers are not yet ready for routine clinical application.

CRP: C-reactive protein; IL-6: interleukin-6; IL-33: interleukin-33; IgE: immunoglobulin E; IgA: immunoglobulin A; IgG: immunoglobulin G; 16S rRNA: 16S ribosomal ribonucleic acid; FeNO: fractional exhaled nitric oxide; VOCs: volatile organic compounds; SP-D: surfactant protein-D; CC-16: club cell secretory protein-16; sRAGE: soluble receptor for advanced glycation end products; VEGF: vascular endothelial growth factor; Pi10: square root of the wall area of a theoretical airway with a 10-mm internal perimeter; TNF-α: tumor necrosis factor-alpha; COPD: chronic obstructive pulmonary disease.

Key Insight: Biomarkers with high validation (CRP, IL-6, SP-D, CT-based markers) can be prioritized for immediate clinical integration, while multi-omics and breathomics remain research tools.

**Table 2.** Ranking of biomarkers by clinical applicability.

Clinical applicability	Biomarkers	Rationale
Routine care (high readiness)	CRP, IL-6, fibrinogen, SP-D, and CT-based metrics (PRM, mucus plugs)	These biomarkers have been validated in large-scale studies such as IMPACT and ECLIPSE. They have standardized assays and are clinically actionable, supporting phenotype-driven management across diverse patient populations.
Near-term translation (moderate readiness)	FeNO, IgE, IgA, sputum cultures, sRAGE, VEGF	These biomarkers have demonstrated potential for patient phenotyping and guided therapy. However, they require additional validation and evidence of cost-effectiveness before being widely implemented in clinical practice.
Research-only (experimental)	VOCs, breathomics, 8-isoprostane, multi-omics (proteomics, metabolomics, virome)	These biomarkers remain in the early experimental stage and currently lack sufficient prospective validation for routine clinical integration. Ongoing research is focused on data harmonization and the development of large-cohort pilot studies to establish their clinical utility.

CRP: C-reactive protein; FeNO: fractional exhaled nitric oxide; IL-6: interleukin-6; IgE: immunoglobulin E; IgA: immunoglobulin A; PRM: parametric response mapping; SP-D: surfactant protein-D; sRAGE: soluble receptor for advanced glycation end products; VEGF: vascular endothelial growth factor; VOCs: volatile organic compounds.

Takeaway: Clinicians should prioritize inflammatory (CRP/IL-6) and serum biomarkers (SP-D) for immediate phenotype-driven decisions, while reserving omics-based biomarkers for research or specialized centers.

## Toward a multi-biomarker panel

Transitioning from isolated biomarker analysis to integrated multi-biomarker panels represents a pivotal step toward precision COPD management.^11,19,22,26^ A clinically meaningful panel must reflect multiple biological and environmental pathways encompassing systemic inflammation, epithelial injury, airway structure, oxidative stress, and microbial dynamics. Combining markers such as CRP, IL-6, fibrinogen, SP-D, and CC-16 with imaging indices like PRM and mucus-plug quantification enables stratification of patients into reproducible inflammatory structural endotypes.^11,19,20,23,24,28,30–32^ Parallel measurement of FeNO, 8-isoprostane, and exposure indices enhances phenotyping of environmentally driven COPD, particularly in biomass-exposed populations.^6,8,49–51^

For clinical adoption, these biomarker panels must undergo analytical validation, standardization, and cost-effectiveness assessment.^57–60^ Inter-laboratory variability in eosinophil counts, cytokine assays, and proteomic signatures currently limits cross-study comparability.^22,33,57^ Harmonizing platforms and defining threshold values for each marker will be essential for regulatory qualification. Composite indices such as the Inflammation Structure Environment (ISE) score, integrating CRP, SP-D, PRM, and oxidative markers, are under evaluation in multicenter cohorts for exacerbation prediction and therapeutic guidance.^22,33,34,54^

AI and machine learning play a central role in panel optimization. AI-based clustering algorithms can identify novel biomarker combinations predictive of specific outcomes such as corticosteroid responsiveness or rapid lung-function decline.^33,34,54,55^ Integration of these predictive models into electronic health records (EHRs) enables real-time clinical decision support, recommending therapy escalation or de-escalation based on patient-specific biomarker trajectories.^57,58^

Equity considerations remain critical. Tiered implementation strategies may deploy simplified blood-based panels (eosinophils, CRP, IL-6) in primary care settings, while advanced molecular and imaging assays can be reserved for tertiary centers.^38,57^ Such scalable models ensure accessibility in LMICs without compromising diagnostic precision. Ultimately, developing a validated, modular multi-biomarker panel offers the most pragmatic pathway to translate mechanistic insight into personalized COPD care at a population level.

A structured decision framework summarizing how these biomarkers guide therapy escalation or de-escalation is illustrated in Figure 1. Novel biomarkers like IgA (humoral immunity) and virome profiles enhance infection-driven exacerbation detection.

**Figure 1.**
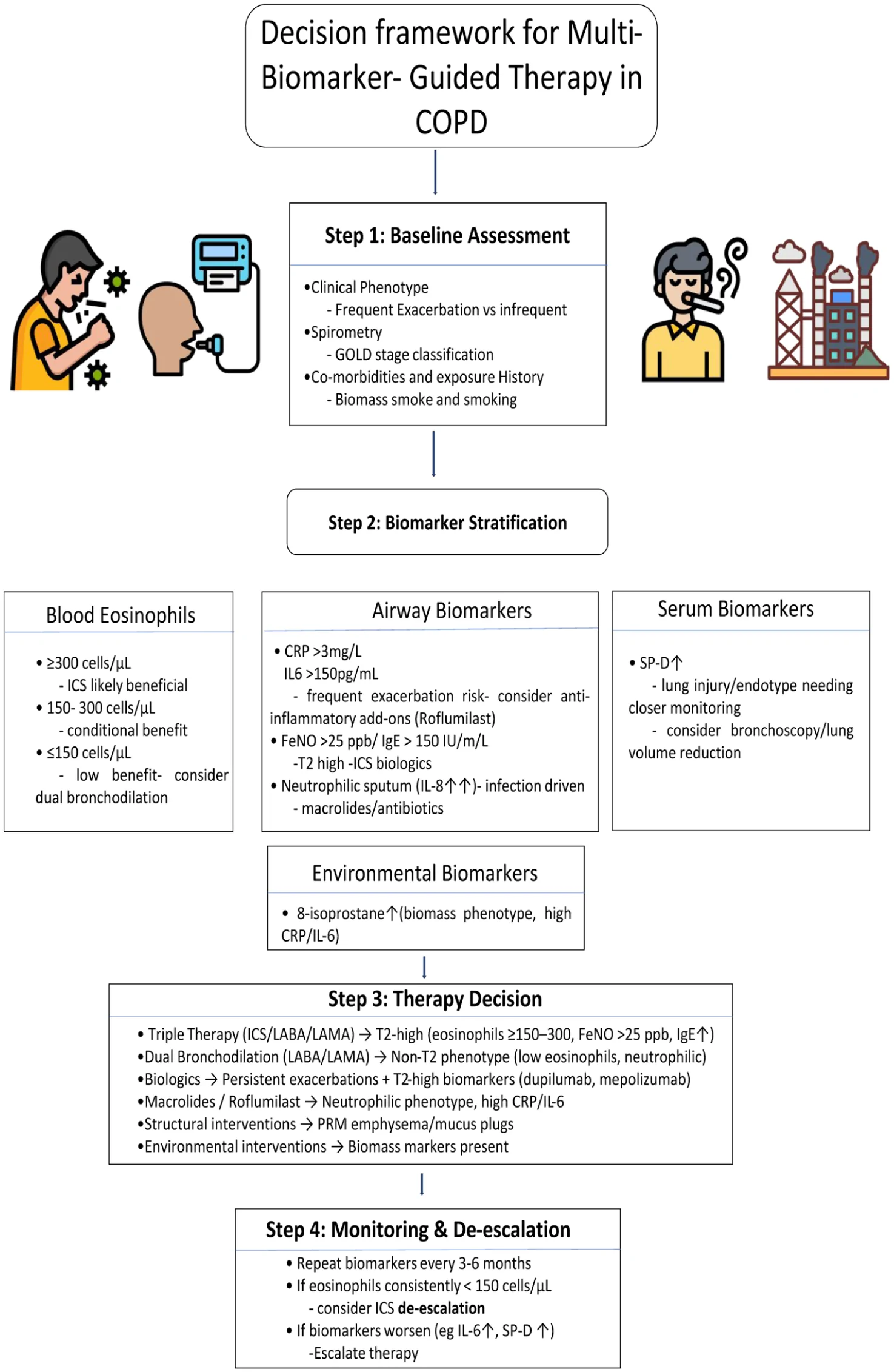
Biomarker-guided COPD therapy algorithm. Therapeutic options shown are intended to represent biomarker-informed considerations and include both guideline-supported and research-phase strategies; they are not intended to imply prescriptive or routine clinical decision-making. Source: The figure is an original illustration created by the authors using Microsoft PowerPoint; icons were sourced from Flaticon and used in compliance with applicable free-use licensing requirements. COPD: chronic obstructive pulmonary disease.

## Therapeutic implications and personalized approaches

The transition from conventional stepwise therapy to biomarker-guided precision management is reshaping COPD care. Traditionally, treatment escalation has relied on symptom burden and exacerbation frequency; however, these clinical indicators fail to reflect the underlying molecular and structural heterogeneity that drives variable responses to therapy.^9–11,18^ Integrating biomarker data enables patient stratification based on distinct inflammatory or structural endotypes, improving therapeutic targeting and minimizing adverse effects.^19,22,26^

### Refining corticosteroid use

Eosinophil-guided ICS therapy remains the cornerstone of anti-inflammatory management in COPD, supported by large randomized trials demonstrating greater ICS efficacy in patients with eosinophil counts ⩾150–300 cells/µL.^2,9,10,14,61,62^ Nonetheless, corticosteroid benefit diminishes in individuals with predominantly neutrophilic or infection-driven inflammation.^18–21,26,63^ Incorporating systemic biomarkers such as CRP, IL-6, and fibrinogen alongside eosinophils allows a more nuanced identification of patients likely to respond to ICS or biologic agents targeting IL-5 and IL-4/13 pathways.^64–66^ Conversely, patients with elevated neutrophil-associated cytokines or bacterial colonization may benefit from non-steroidal anti-inflammatory approaches, such as macrolides, phosphodiesterase-4 inhibitors, or statins.^20,21,40,41^

### Targeting non-T2 inflammation

Non-eosinophilic COPD, characterized by neutrophilic and macrophage-predominant inflammation, remains a therapeutic challenge. Recent studies have explored IL-6 receptor blockade, TNF-α inhibitors, and IL-33/ST2 pathway modulation as potential anti-inflammatory strategies in this endotype.^64–66^ TNF-α is a key mediator of neutrophilic airway inflammation and systemic inflammatory burden in COPD; however, clinical trials of TNF-α inhibitors, such as infliximab and etanercept, in unselected COPD populations failed to demonstrate consistent clinical benefit and were associated with increased infection risk.^67,68^ These findings indicate that TNF-α represents a biologically relevant but currently non-actionable therapeutic target without biomarker-guided patient selection. Early-phase trials of anti–IL-33 monoclonal antibodies demonstrate improvements in airway neutrophilia and symptom burden, although larger studies are required to confirm clinical benefit.^65,66^ Multi-biomarker panels incorporating IL-6, SP-D, and PRM metrics may help identify candidates for such targeted biologics, particularly in research and early translational settings.^15,34,54,58,59,62,63^

### Structural and imaging-guided interventions

Quantitative imaging provides actionable insights beyond lung-function decline. PRM and mucus-plug quantification identify patients at risk of rapid disease progression and exacerbations, guiding more aggressive pharmacologic or interventional strategies.^20,24,30–32^ In advanced emphysema with heterogeneous destruction, endobronchial valve therapy or lung-volume reduction procedures may improve symptoms and survival.^20,24^ Combining imaging-derived indices with inflammatory biomarkers can refine the timing of such interventions and monitor their physiologic impact.^8,11,20,24^

### Digital and AI-driven personalization

AI and predictive analytics now support individualized treatment optimization by integrating biomarker trends, imaging data, and clinical history.^22,33,34,54,55^ Machine-learning models have been shown to predict exacerbations, hospitalization risk, and therapeutic response with greater accuracy than traditional indices.^33–36^ Embedding these algorithms into EHRs allows clinicians to receive real-time recommendations for therapy escalation or de-escalation based on evolving patient profiles.^57,58^ Such tools enhance proactive management and enable continuous learning across populations.

### Equity and implementation

Ensuring equitable implementation of precision COPD care remains essential, particularly in LMICs where biomass exposure and pollution-driven COPD predominate.^6,8,49–51^ Tiered diagnostic models using simple biomarkers (eosinophils, CRP, and IL-6) for primary care and advanced panels (SP-D, PRM, 8-isoprostane) for tertiary centers can achieve scalability and cost-efficiency.^38,57^ Integrating these frameworks into community health systems, coupled with portable diagnostic devices and telemedicine platforms, will be key to global adoption.

## Clinical and policy implications: outcomes, challenges, trials, global health, and research priorities

The transition toward multi-biomarker-based precision medicine in COPD marks a fundamental shift from population-level algorithms to individualized, data-driven care. Integrating molecular, structural, and environmental biomarkers allows earlier identification of disease activity, refined therapy selection, and more accurate outcome prediction. Clinical studies increasingly demonstrate that biomarker-guided management reduces exacerbation frequency, minimizes corticosteroid overuse, and improves survival and quality of life.^2,12,19,22^ Real-world evidence also indicates improved adherence and reduced hospitalization when therapy is guided by validated biomarker profiles rather than symptom-based assessment alone.^12,19^

Multi-domain biomarker frameworks encompassing systemic inflammation (CRP, IL-6, fibrinogen), epithelial damage (SP-D, CC-16), structural remodeling (PRM, mucus plugs), and oxidative stress (8-isoprostane) offer a multidimensional understanding of COPD pathobiology.^11,19,20,23,24,28,30–32^ Incorporating these parameters into clinical workflows enables more accurate prediction of exacerbations and individualized treatment planning. Large-scale initiatives such as COPDGene-III, SPIROMICS-2, and SUMMIT are validating integrated biomarker signatures for prognostic precision and therapeutic optimization.^22,33,34,54,55^ Findings from these studies are expected to inform future GOLD recommendations and biomarker qualification guidelines.

Despite encouraging progress, several challenges hinder clinical translation. These include assay variability, cost constraints, limited interoperability across platforms, and a lack of biomarker standardization.^22,33,57^ Establishing certified reference laboratories, harmonized calibration protocols, and quality-assurance networks will be essential for reproducibility and regulatory endorsement. Embedding biomarker data into EHRs with automated decision-support algorithms could enhance clinician adoption and ensure consistent care delivery.^57,58^ Furthermore, updated reimbursement and policy frameworks are required to recognize the long-term value of precision diagnostics in reducing healthcare utilization and hospital costs.

Equity remains a central pillar of implementation. In LMICs, where biomass combustion and pollution are predominant risk factors, eosinophil-based thresholds frequently misclassify disease severity.^6,8,49–51^ Portable point-of-care biomarker tools, coupled with simplified diagnostic panels, can extend access to precision care at the community level. Tiered diagnostic systems employing basic inflammatory markers in primary care and advanced molecular or imaging biomarkers in tertiary centers offer a scalable and sustainable model.^38,57^ Collaborative efforts between governments, academic institutions, and industry partners will be essential to ensure equitable access and training.

## Gaps in knowledge and future directions

Biomarker research in COPD remains limited because most studies involve small, heterogeneous cohorts and lack standardized analytical methods. Several promising biomarkers, including SP-D, CC-16, and oxidative-stress markers, still do not have validated reference ranges or established clinical thresholds. The integration of imaging, multi-omics, and exposomic data into clinical workflows remains experimental, and real-world validation across diverse populations is scarce. Accordingly, many of these emerging approaches should currently be regarded as research-phase or early translational tools rather than ready for routine clinical implementation. In addition, economic evidence supporting large-scale biomarker testing and clinician familiarity with interpreting multi-biomarker panels is insufficient.

Future research should prioritize large longitudinal validation studies, harmonization of laboratory and imaging standards, and establishment of clinically meaningful biomarker cutoffs. Integrating multi-omics and radiomics with AI could enable real-time monitoring and individualized risk prediction.^22,34,54,55^ Adaptive biomarker-driven clinical trials and cost-effective diagnostic platforms will be essential for implementation.^64–66^ Embedding precision-medicine principles into everyday COPD care can transform scientific innovation into global clinical benefit.

## Conclusion

The evolution of COPD management from uniform treatment algorithms to biomarker-guided precision medicine represents a transformative milestone in modern respiratory care. Multi-biomarker frameworks that integrate inflammatory, structural, microbial, and environmental domains provide a comprehensive understanding of disease heterogeneity that extends beyond eosinophil-based stratification. These integrative models enable clinicians to predict exacerbations more accurately, individualize therapeutic strategies, and reduce unnecessary corticosteroid exposure while ensuring equitable and cost-effective healthcare delivery. The incorporation of AI and digital technologies into clinical workflows will allow real-time interpretation of biomarker data, thereby enhancing clinical decision-making and disease monitoring. Successful implementation will require international collaboration to standardize biomarker assays, validate predictive models, and ensure affordability and accessibility in low- and middle-income settings. By aligning scientific innovation with health-system reform, multi-biomarker precision medicine has the potential to redefine COPD diagnosis, prognosis, and treatment, ultimately improving health outcomes and advancing global respiratory care.

## Supplemental Material

sj-pdf-1-taj-10.1177_27558428261452294 – Supplemental material for Advancing chronic obstructive pulmonary disease management: The case for multi-biomarker precision medicine—A narrative review Supplemental material, sj-pdf-1-taj-10.1177_27558428261452294 for Advancing chronic obstructive pulmonary disease management: The case for multi-biomarker precision medicine—A narrative review by Anusha Sundararaman, Thangavel Mahalingam Vijayakumar and Nalini Jayanthi Nagesh in Therapeutic Advances in Chronic Disease

## Data Availability

Not applicable.
